# Relationship Between the Provision of Injection Services in Ambulatory Physician Offices and Prescribing Injectable Medicines

**Published:** 2017

**Authors:** Naeimeh Yousefi, Arash Rashidian, Fatemeh Soleymani, Abbas Kebriaeezade

**Affiliations:** a *Faculty of Pharmacy, Tehran University of Medical Sciences, Tehran, Iran. *; b *Department of *Health Management and Economic*, School of Public Health, Tehran University of Medical Sciences, Tehran, Iran. *; c *Knowledge Utilization Research Center, Tehran University of Medical Sciences, *; d *Tehran, Iran. Department of Pharmacoeconomics and Pharmaceutical Management, Faculty of Pharmacy, Tehran University of Medical Sciences, Tehran, Iran. *; e *Iran food and drug organization. MOH&ME Iran, Tehran and National Committee on Rational Drug Use, Food and Drug Organization, Ministry of Health and Medical Education. *; f *Department of Toxicology and Pharmacology, Faculty of Pharmacy and Pharmaceutical Sciences Research Center, Tehran University of Medical Sciences, Tehran, Iran. *; g *Department of Toxicology and Pharmacology, Faculty of Pharmacy and Pharmaceutical Sciences Research Center, Tehran University of Medical Sciences, Tehran, Iran. *; h *Department of Pharmacoeconomics and Pharmaceutical Management, Faculty of Pharmacy, Tehran University of Medical Sciences, Tehran, Iran.*

**Keywords:** rational use of medicines, injection medicines, survey, developing countries, outpatient care

## Abstract

Overuse of injections is a common problem in many low-income and middle income countries. While cultural factors and attitudes of both physicians and patients are important factors, physicians› financial intensives may play an important role in overprescribing of injections. This study was designed to assess the effects of providing injection­ services in physicians› ambulatory offices on prescribing injectable medicines. This cross-sectional study was conducted in Tehran in 2012 -2013and included a random sample of general physicians, pediatricians and infectious disease specialists. We collected data on the provision of injection services in or in proximity of physician offices, and obtained data from physicians› prescriptions in the previous three-month period. We analyzed the data using ANOVA, Student›s t-test and linear regression methods. We obtained complete data from 465 of 600 sampled physicians. Overall 41.9% of prescriptions contained injectable medicines. 75% of physicians offered injection services in their offices. Male physicians and general physicians were more likely to offer the services, and more likely to prescribe injectables. We observed a clear linear relationship between the injection service working hours and the proportion of prescriptions containing injectables (p-value<0.001). Providing injection service in the office was directly linked with the proportion of prescriptions containing injectables. While provision of injection services may provide a direct financial benefit to physicians, it is unlikely to be able to substantially reduce injectable medicines› prescription without addressing the issue.

## Introduction

Irrational use of medicines still remains a serious issue in low-income and middle-income countries. Irrational use of medicines results in potential harms to population health (1).It also results in increasing pharmaceutical expenditures, resulting in governments struggle to keep such costs under control (2-4). Despite a long history of establishing rational use of medicines in many countries, including in Iran, the problem still persists (5). Despite these challenges, and the importance of improving irrational use of medicines (6), little evidence exists that assesses the health service organization aspects that might affect inappropriate prescribing (7).

Among different type of inappropriate uses of medicines, overuse of injectable medicines not only wastes the health care resources, it also increases risks of adverse effects for patient (8). Also the injection is an expensive process and needs skilled health care provider input, further the costs that are imposed on households and payers (including insurance organizations). It has been argued that use of injectable medicines is no warranted in the vast majority of patients visited at ambulatory settings (9). Still injectable medicines are frequently prescribed in ambulatory settings in many low-income and middle-income countries, including in Iran (10, 11). 

In this study we assessed whether the existence of an injection service facility within or in the near vicinity of the physician office affects his or her prescribing of injectable medicines. 

## Methods

*Study design*

We conducted a cross-sectional survey, alongside a randomized-controlled trial which assessed effectiveness and cost – effectiveness of audit and feedback on physicians’ prescribing indicator (5).

*Population and setting*

General physicians, pediatricians and infectious diseases specialists with ambulatory offices in Tehran and a minimum of 100 prescriptions per month who had active contracts with at least one of the main social health insurance organizations were eligible for inclusion in the study (5). 600 physicians were randomly selected from the list of eligible physicians, and their office address and telephone numbers were obtained from the insurance organizations. 

*Data sources and analysis*

We used the physicians baseline prescribing, collected for use a randomized controlled trial, in this study. Additionally we collected data on the existence of injection service facilities within or adjacent to the physician office via a telephone survey of the physicians› offices. We asked about the working hours, the professional status of the injection service provider (physician or injection technician) and the location of the facility. 

**Figure 1:**
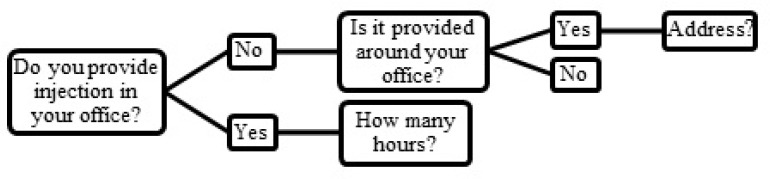
Questions about injection service within or adjacent to physicians› offices.

Figure 1. shows the procedures that were followed while asking about the injection service.

Univariate statistical analysis was conducted using Student›s t-test and one-way ANOVA tests. Correlation analysis was used to evaluate the relationship between working hours and average percentage of injectable medicines. Multiple linear regressions were used to conduct the multivariate analysis. 

*Ethical approval*

All physicians’ related data were anonymized before analyses. We received research ethics committee approval from the Pharmaceutical Sciences Research Center of TUMS Ethics Committee Registration Number: 90-02-27-07.

## Results

A total of 600 physicians were sampled for this study and 465 valid answers were obtained from the telephone survey. The valid response rate was 77.5%. About half (47%) of the non-responses were due to errors within physicians› contact data: wrong recorded phone number (38), replaced office location (25). The remaining 72 physicians (53% of non-response) did not answer the calls.

84% of general physicians and 40% of specialists provided injection services in their offices. Female physicians were less likely to provide such services. More information is available in Table 2.

Univariate analyses suggested that physicians who provided injection services within their offices (whether by physician or by injection technician) were more likely to prescribe injectable medicines than those who were not (48% versus 28%; p-value<0.001). Among physicians that had no injection services within their offices, existence of an injection service in the vicinity of the physician office did not seem to result in a statistically significant difference in prescribing injectable medicines (p-value = 0.27). A lower proportion of female physicians and specialists’ prescriptions contained an injectable medicine as compared with their male counterparts (33% versus 45% of prescriptions, respectively; p-value < 0.001). General physicians were also more likely to issue prescriptions containing injectable medicines than the specialists groups under study. 

The assessment of relationship between injection service status and demographic characteristics, revealed that male physicians and general practitioners were more likely to provide injection services (R^2^ = 0.26 and P-value < 0.001).

**Figure 2:**
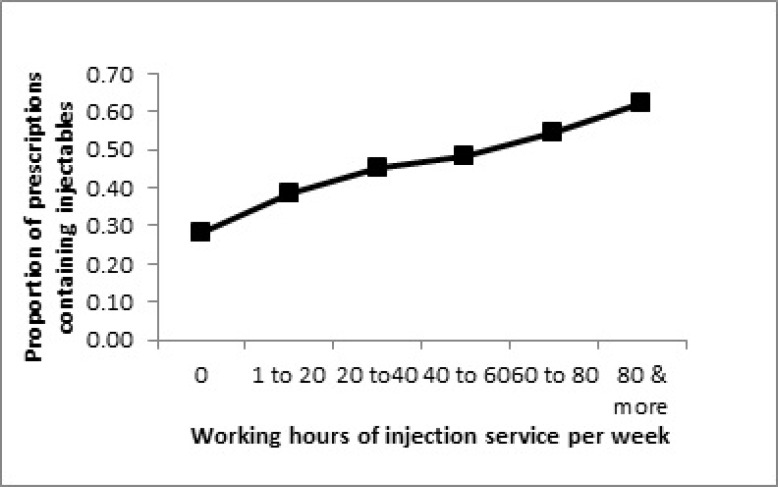
Relationship between injection service working hours and prescription of injection medicines.

As it is shown in Figure 2, there is a linear relationship between the injection services working hours per week and prescription of injectable medicines. While those with no provision of injection services prescribed injectable medicines in less than 30% of their prescriptions, the physicians that offered injection services for about 80 h per week, prescribed injectables in over 60% of their prescriptions.

## Discussion

Our study physicians working in ambulatory offices in Tehran suggests that there is a strong relationship between the gender, specialty of physicians and the provision on injection services and the proportion of physician›s prescriptions containing injectable medicines. Among these, the provision of injection services seems to have a linear relationship with prescribing injectable medicines.We found that female physicians were less likely to issue prescriptions containing injectable medicines (12% less such prescription as compared with male physicians). This finding was in contrast with a previous study by Wang *et al*. who observed that physician gender had no significant impact on injectable medicines prescribed (12). At the same time our findings about the effect of specialty on physicians’ prescription behavior is observed in other studies (5, 13).

**Table 1:** The characteristics of the sample and respondents

	All	Female (%)	Male (%)	Respondents (%)	Within respondents	
					Female	Male
All the physicians	600	128 (21.3)	472 (78.7)	465 (77.5)	95 (20.4)	370 (79.6)
General physicians	519	106 (20.4)	413 (79.6)	418 (80.5)	82 (19.6)	336 (80.4)
Pediatrician & infectious disease specialists	81	22 (27.2)	59 (72.8)	47 (58.0)	13 (27.7)	34 (72.3)

**Table 2:** Characteristics of injection facilities within or nearby the physicians' offices

	Within physician office (%)		Nearby physician office (%)	No injection facility (%)
	By physician	By injection technician		
GPs Male Female	(8)34 (9) 31 (4) 3	(75)314 (76) 255 (72)59	30 (7) (7)24 (7)6	40 (10) 26 (8) 14 (17)
specialists Male Female	(6) 3 (9) 3 0	(34) 16 (32) 11 (39)5	(26)12 (29.5) 10 (15)2	(34)16 (29.5) 10 (46) 6

As study limitations, our study was limited to physicians in Tehran, so this might limit its application to other settings in Iran, and in other countries. Also we relied upon self-report of physicians› offices secretaries about the injection service characteristics. Ideally such data should have been collected via observation of the services. However, as the service is part of the package of care the physicians provide, it is unlikely that the mode of data collection have biased the study findings in a significant way. 

However we found no previous study that had assessed the linkage between the provision of the injection services and its impact on inappropriate prescribing of injectable medicines in ambulatory care. While high usage of injectable medicines is a main concern in many low-income and middle-income countries, and in the Eastern Mediterranean Region in particular (7), our findings suggest the organization of ambulatory care might be an important factor to affect such prescriptions. 

## Conclusions

Reducing the amount of injectable medicines used in ambulatory care is an important policy objectives toward a more rational use of medicines. Our findings suggest the organization of ambulatory care is an important determinant of such behavior. We observed that physicians, who have provide injection services in their offices, prescribe more injection medicines. In a country like Iran, where physician offices are highly regulated moving towards limiting such services in ambulatory care may have a clear impact on improving appropriate use of medicines. 
